# Use of tobacco and e-cigarettes among youth in Great Britain in 2022: Analysis of a cross-sectional survey

**DOI:** 10.18332/tid/156459

**Published:** 2023-01-21

**Authors:** Parris J. Williams, Hazel Cheeseman, Deborah Arnott, Laura Bunce, Nicholas S. Hopkinson, Anthony A. Laverty

**Affiliations:** 1 National Heart and Lung Institute, Imperial College London, London, United Kingdom; 2 Action on Smoking and Health, London, United Kingdom; 3 School of Public Health, Imperial College London, London, United Kingdom

**Keywords:** e-cigarettes, youth tobacco use, Great Britain

## Abstract

**INTRODUCTION:**

Although e-cigarettes can be an effective form of nicotine substitution for adults attempting to quit smoking, their use among children and young people is a concern. Accurate data about this are needed to inform debates over policy and regulation in the UK and elsewhere.

**METHODS:**

Using data from an online survey of 2613 youth aged 11–18 years, conducted by the market research company YouGov in March 2022, we present prevalence estimates of e-cigarette and tobacco use. We use logistic regression models to assess differences in e-cigarette use, tobacco use and use of disposable e-cigarettes across a range of covariates including age, sex, tobacco smoking status, social class, and country.

**RESULTS:**

Among the 18.0% of those surveyed who reported ever having smoked a cigarette, 83.9% were not regular (at least once per week) smokers and 16.1% were (15.1% and 2.9% of the total sample, respectively). Among the 19.2% of those surveyed who had ever used an e-cigarette, 79.2% were not regular users, while 20.8% were (15.2% and 4.0% of the total sample, respectively). Regular e-cigarette use was more common than regular tobacco smoking (4.0% vs 2.9%). E-cigarette use was more common among those who also smoked tobacco, with 9.0% of never e-cigarette users ever smoking tobacco, compared with 89.4% of regular e-cigarette users. Both smoking and e-cigarette use were associated with increasing age and use by others within the home, but not with social class. Use of disposable e-cigarettes was reported by 53.8% of those who have ever used an e-cigarette, and more common among females than males.

**CONCLUSIONS:**

Regular e-cigarette use is now more common than smoking in children and youth, though the majority of this is among those who have also smoked tobacco. Measures to reduce the appeal of both e-cigarettes and tobacco to children and young people are warranted.

## INTRODUCTION

The use of e-cigarettes as a tool for harm reduction among people with tobacco dependence has gained recent momentum in the UK, and in 2021 the Medicines and Healthcare Products Regulatory agency (MHRA) announced that it would facilitate the process for e-cigarettes to become licensed as medicinal products. This would allow them to be marketed explicitly as smoking cessation aids and prescribed in smoking cessation clinics and elsewhere^1^. Although UK youth smoking rates are at an all-time low^2^, there are growing concerns that e-cigarette use may be developing among tobacco-naive young people, particularly the suggestion that e-cigarette use within this population may act as a gateway to cigarette smoking. In the US, a 2018 report by the National Academies of Sciences, Engineering, and Medicine (NASEM) concluded that there was evidence for a causal effect of e-cigarette use on youth transition from non-smoker to ever smoker, while the Centers for Disease Control and Prevention (CDC) describe youth e-cigarette use as a serious public health concern^3,4^. Within the UK, a 2020 PHE report stated that the majority of youth never smokers had also never used an e-cigarette with only 0.8–1.3% of tobacco-naive youth reporting that they were current e-cigarette users, concluding with a statement that e-cigarette prevalence among the UK’s young people needs to be closely monitored^5^.

Given the importance of observing smoking and e-cigarette use within the younger population and the need for vigilance to track developments in the market, we report data from the 2022 ASH/YouGov Smoke-free GB-Youth survey on youth smoking and e-cigarette use.

## METHODS

### Data

Data came from the ASH/YouGov Smoke-free GB-Youth survey of 2613 youth aged 11–18 years, collected between 1 and 29 March 2022. Data were collected by YouGov, a public limited company, from an online panel recruited using proprietary software which is detailed elsewhere^6^. Participants are recruited to a main online panel and give sociodemographic information which is periodically updated. From this main online panel a method called Active Sampling assesses which members from the main panel are suitable for specific data collections, and this study is based on members contributing to the Smoke-free GB-Youth Survey^7^. The survey is designed to be representative of the population and survey weights are derived by YouGov in order to make the data representative across age, sex and region. Consent was provided by participants if they were aged ≥16 years, and by their parents if aged <16 years.

### Measures

E-cigarette use was assessed using the question ‘Which one of the following is closest to describing your experience of e-cigarettes?’. We categorized this into never e-cigarette users (‘I have never used an e-cigarette’), non-regular e-cigarette users (‘I have only tried an e-cigarette once or twice’; ‘I used e-cigarettes in the past but no longer do’; ‘I use e-cigarettes more than once a month, but less than once a week’; ‘I use e-cigarettes sometimes, but no more than once a month’), and regular use (‘I use e-cigarettes more than once a week but not every day’; ‘I use e-cigarettes every day’). We also classified respondents who replied that they had never heard of e-cigarettes as never e-cigarette users. Tobacco use was assessed using the same categories. We present regression analyses for both regular use and ever use of both tobacco and e-cigarettes.

Participants who reported ever use of e-cigarettes were also asked about the order of product use. We report the percentage of cigarette smoking among those choosing to select ‘I tried smoking a real cigarette before I first tried using an e-cigarette’ compared with those who selected ‘I tried using an e-cigarette before I first tried smoking a real cigarette’. Ever e-cigarette users were also asked: ‘Have you ever used a disposable electronic cigarette (non-rechargeable)?’ which was classified as yes or no.

Other data included were age group (11–13, 14–15, 16–17, and 18 years), sex, social class [based on the Registrar General classification of occupations and classified as ABC1 (higher) vs C2DE (lower)], and country of Great Britain (England, Scotland, Wales). We assessed household use of e-cigarettes and tobacco from the questions: ‘Does anyone who currently lives in your home use an e-cigarette? (yes/no)’, and ‘Who in your family, if anyone, smokes tobacco cigarettes at the moment?’. For cigarette use in the family, we created a binary variable which categorized all of: ‘mother (or female carer)’, ‘father (or male carer)’, ‘brother or sister’, ‘grandparent’ and ‘other family member(s)’ as yes. We also assessed exposure to e-cigarette promotion using the question: ‘For the following question, by 'being promoted', we mean something that tries to increase interest in or demand for e-cigarettes. In which, if any, of the following places do you ever see e-cigarettes being promoted?’. Participants could select either none, or more than one response, and we categorized exposure as selecting at least one of billboards, shops, online, on buses, on TV, in newspapers/magazines, or ‘somewhere else’. We did not have data on exposure to tobacco advertising.

### Statistical analysis

Descriptive statistics are presented after accounting for survey weighting. Logistic regression analyses assessed differences in ever and regular e-cigarette and tobacco use and use of disposable e-cigarettes among those who reported ever use of e-cigarettes. All these models were adjusted for the sociodemographic factors age, sex, country (England, Scotland, Wales) and social class. Models of e-cigarette use were also adjusted for reporting seeing e-cigarette promotion as well as e-cigarette use within the home. Models of tobacco smoking were adjusted for sociodemographic factors as well as family tobacco smoking. Analyses were performed in Stata 15.

## RESULTS

The majority of participants lived in England (2281), with 206 in Scotland and 126 in Wales (Table 1). There were 1756 participants in higher social classes and 755 in lower social classes. There were 341 participants aged 18 years and the majority of participants (997) were aged 11–13 years.

**Table 1:** Analytic sample, numbers weighted using survey weighting. Data from 2022 survey of youth aged 11–18 years in Great Britain

*Characteristics*	*n*	*%*
**Age** (years)		
11–13	997	38.2
14–15	625	23.9
16–17	649	24.8
18	341	13.1
**Sex**		
Male	1340	51.3
Female	1273	48.7
Social class*		
Higher	1756	67.2
Lower	755	28.9
**Country**		
England	2281	87.3
Scotland	206	7.9
Wales	126	4.8
**Overall**	2613	100

* Defined by Registrar General’s classification of occupations and classified as ABC1 (higher) versus C2DE (lower).

Ever smoking tobacco was reported by 18.0% (417) of the sample; 83.9% (350) of these were non-regular smokers (absolute percentage 15.1%), and 16.1% (67) were regular smokers (absolute percentage 2.9%) (Table 2). Among the 19.2% (445) of those surveyed who had ever used an e-cigarette, 79.2% were not regular users, while 20.8% were (15.2% and 4.0% respectively). Regular e-cigarette use was more common than regular tobacco smoking (4.0% vs 2.9%). E-cigarette use was more common among those who also smoked tobacco, with 9.0% of never e-cigarette users ever smoking tobacco, compared with 89.4% of regular e-cigarette users.

**Table 2:** E-cigarette use (%) by tobacco smoking status among youth aged 11–18 years in Great Britain, 2022 (N=2318)

*Characteristics*	*Never smoked tobacco % (n)*	*Non-regular tobacco smoker % (n)*	*Regular smoker % (n)*	*Ever smoked tobacco % (n)*	*Total % (n)*
Never used e-cigarettes	91.0 (1704)	8.5 (159)	0.5 (10)	9.0 (169)	80.8 (1873)
Non-regular e-cigarette user	24.4 (86)	57.9 (204)	17.8 (63)	75.65 (267)	15.2 (352)
Regular e-cigarette user	10.6 (10)	41.2 (38)	48.2 (45)	89.4 (83)	4.0 (93)
Ever e-cigarette user	21.5 (96)	54.4 (242)	24.1 (107)		19.2 (445)
Total	82.0 (1901)	15.1 (350)	2.9 (67)	18.0 (417)	100 (2318)

Never: ‘I have never used an e-cigarette’. Non-regular: ‘I have only tried an e-cigarette once or twice’, ‘I used e-cigarettes in the past but no longer do’, ‘I use e-cigarettes more than once a month, but less than once a week’, and ‘I use e-cigarettes sometimes, but no more than once a month’. Regular: ‘I use e-cigarettes more than once a week but not every day’, and ‘I use e-cigarettes every day’. Respondents who said that they had never heard of e-cigarettes were also classified as never e-cigarette users.

Questions on ordering of product use were asked only of those who reported ever trying an e-cigarette. The majority of people who had ever used an e-cigarette had also tried smoking (65.0%; 368); 19.4% (110) reported that they used an e-cigarette before smoking a regular cigarette, and 45.6% (258) reported smoking a regular cigarette before using an e-cigarette. Being a regular smoker was more common among those who reported using tobacco before e-cigarettes than among those who used e-cigarettes first (20.2% vs 9.3%).

**Figure 1:**
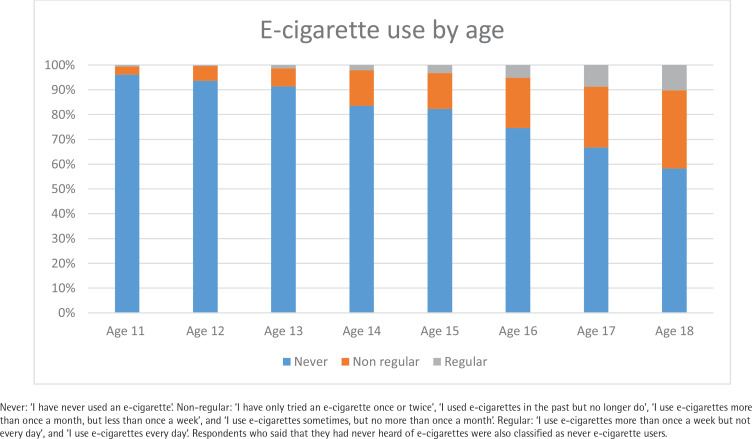
E-cigarette use among those aged 11–18 years, in Great Britain, 2022

Differences in ever and regular use of e-cigarettes are shown in Table 3. Ever use was more common as age increased (p<0.001); participants aged 18 years were considerably more likely to report ever use than those aged 11–13 years (adjusted odds ratio, AOR=13.79; 95% CI: 8.91–21.35) (Figure 1). Ever use was more common among participants reporting regular e-cigarette use in their home (AOR=6.65; 95% CI: 5.08–8.70), although no differences were detected by social class or country. Associations were similar for regular use, with use more common among older participants and those reporting e-cigarette use inside the home (AOR=20.75; 95% CI: 11.86–36.28). Reporting exposure to e-cigarette promotion was linked to greater likelihood of ever use of e-cigarettes (AOR=1.61; 95% CI: 1.23–2.50) with a weaker association with regular e-cigarette use (AOR=1.52; 95% CI: 0.93–2.50).

**Table 3:** Ever and regular use of e-cigarettes by selected characteristics among youth aged 11–18 years in Great Britain, 2022 (N=2003)

*Characteristics*	*Ever use*		*Regular use*	
	*AOR*	*95% CI*	*AOR*	*95% CI*
**Gender**				
Boys (Ref.)	1		1	
Girls	1.11	0.87–1.41	1.25	0.81–1.92
**Age** (years)				
11–13 (Ref.)	1		1	
14–15	3.23	2.09–4.99	2.80	1.00–7.84
16–17	7.93	5.23–12.00	9.54	3.65–24.94
18	13.79	8.91–21.35	10.68	4.08–27.93
**Country**				
England (Ref.)	1		1	
Scotland	1.23	0.85–1.78	1.02	0.52–2.02
Wales	0.52	0.25–1.10	0.40	0.08–1.98
**Social class**				
Higher* (Ref.)	1		1	
Lower	0.93	0.72–1.20	0.71	0.44 – 1.13
**Awareness of e-cigarettes**				
Has not seen e-cigarettes promoted**	1		1	
Has seen e-cigarettes promoted	1.61	1.23–2.09	1.52	0.93–2.50
**E-cigarette use**				
No e-cigarette use in home (Ref.)	1		1	
E-cigarette use in home	6.65	5.08–8.70	20.75	11.86–36.28

AOR: adjusted odds ratio.

* Defined by Registrar General’s classification of occupations and classified as ABC1 (higher) versus C2DE (lower).

** In at least one of billboards, shops, online, on buses, on TV, in newspapers/magazines, or ‘somewhere else’.

Differences in ever and regular tobacco smoking are shown in Table 4. These present a similar picture to that for e-cigarette use. Ever use was more common among older participants (e.g. AOR for youth aged 18 years vs youth aged 11–13 years = 10.79; 95% CI: 7.43–15.68) as well as those with cigarette smoking in the family (AOR=2.42; 95% CI: 1.93–3.04). Regular smoking was more common among older participants (p<0.001) and those reporting cigarette smoking in the family (AOR=3.87; 95% CI: 2.34–6.41). We did not detect differences by sex or social class for either ever or regular tobacco smoking.

**Table 4:** Ever and regular tobacco smoking by selected characteristics among youth aged 11–18 years in Great Britain, 2022 (N=2474)

*Characteristics*	*Ever use*		*Regular use*	
	*AOR*	*95% CI*	*AOR*	*95% CI*
**Gender**				
Boys (Ref.)	1		1	
Girls	0.92	0.74–1.15	0.84	0.51–1.39
**Age** (years)				
11–13 (Ref.)	1		1	
14–15	2.42	1.62–3.61	1.28	0.47–3.51
16–17	6.50	4.53–9.34	3.80	1.62–8.89
18	10.79	7.43–15.68	5.81	2.47–13.65
**Country**				
England (Ref.)	1		1	
Scotland	1.18	0.84–1.65	1.33	0.67–2.63
Wales	0.51	0.27–0.97	0.61	0.19–1.96
**Social class**				
Higher* (Ref.)	1		1	
Lower	1.01	0.79–1.27	1.11	0.67–1.85
**Cigarette smoking in family**				
No cigarette smoking in family (Ref.)	1		1	
Cigarette smoking in family	2.42	1.93–3.04	3.87	2.34–6.41

AOR: adjusted odds ratio.

* Defined by Registrar General’s classification of occupations and classified as ABC1 (higher) versus. C2DE (lower).

Differences in use of disposable e-cigarettes among ever e-cigarette users are shown in Table 5. Use of disposable products was more common among females (AOR=1.69; 95% CI: 1.14–2.51), aged 18 years (AOR=2.78; 95% CI: 1.17–6.61), and those reporting e-cigarette use in the home (AOR=2.60; 95% CI: 1.74–3.88).

**Table 5:** Use of disposable e-cigarettes among ever e-cigarette users aged 11–18 years in Great Britain, 2022 (N=513)

*Characteristics*	*%*	*AOR*	*95% CI*
**Gender**			
Boys (Ref.)	50.9	1	
Girls	59.3	1.69	1.14–2.51
**Age** (years)			
11–13 (Ref.)	41.1	1	
14–15	45.7	1.29	0.53–3.14
16–17	59.6	2.33	0.99–5.49
18	62.5	2.78	1.17–6.61
**Country**			
England (Ref.)	54.9	1	
Scotland	56.7	0.80	0.45–1.41
Wales	60.3	1.79	0.38–8.4
**Social class**			
Higher* (Ref.)	53.3	1	
Lower	58.8	1.24	0.82–1.89
**Awareness of e-cigarettes**			
Has not seen e-cigarettes promoted** (Ref.)	54.8	1	
Has seen e-cigarettes promoted	56.6	1.22	0.77–1.95
**E-cigarette use**			
No e-cigarette use in home (Ref.)	45.6	1	
E-cigarette use in home	66.3	2.60	1.74–3.88

AOR: adjusted odds ratio.

* Defined by Registrar General’s classification of occupations and classified as ABC1 (higher) versus C2DE (lower).

** In at least one of billboards, shops, online, on buses, on TV, in newspapers/magazines, or ‘somewhere else’.

## DISCUSSION

Youth tobacco smoking and e-cigarette usage remained low in 2022, with e-cigarette usage in this population now slightly higher than regular tobacco smoking. Compared to data from 2020–2021, the low smoking prevalence is unchanged and regular e-cigarette use is also similar (4.0% vs 4.3%)^8^. Most e-cigarette users reported that they have experimented with or are regular tobacco users alongside their e-cigarette use.

Despite youth smoking rates being at an all-time low, increases in e-cigarette use in those without previous tobacco smoking raises the concern about a potential gateway effect of e-cigarette use to tobacco smoking, which is concerning. Although there are likely to be common susceptibility factors for both tobacco and e-cigarette use (family and peer-group use, social class, genetic susceptibility etc.), there is a need for regulation and enforcement to avoid uptake of e-cigarette use among children. This is necessary both to reduce the risk of potential harms from short- or long-term exposure to vaping, and to avoid any risk of transitioning to smoking. As adult smoking rates decrease, and rates of adult e-cigarette use increase, it is to be expected that patterns of use among young people will reflect this. Thus, in 2018, ASH Smoke-free survey data highlighted that 18.3% of young ever tobacco smokers reported having tried e-cigarettes before they smoked a conventional cigarette for the first time^8^; the same survey 3 years later, this figure had increased to 29.3% of youths trying e-cigarettes before smoking tobacco^8^. Data from different settings are similar. For example, a 2020 longitudinal analysis of e-cigarette and smoking use in the US concluded that adolescents who reported current exclusive use of e-cigarettes were five times more likely to be tobacco smokers one year later than non-current e-cigarette users^9^. A meta-analysis of three longitudinal studies from the UK has identified that ever e-cigarette users were six times more likely to take up tobacco smoking than never e-cigarette users, and there are similar findings from studies across Europe and North America^10,11^.

The present findings indicate that current laws limiting child access to tobacco and e-cigarettes are not adequately enforced and underscore recent recommendations for increased investment in local smoke-free enforcement actions^12^. Current regulation in the UK prohibits the sale of nicotine containing e-cigarettes to those aged <18 years^13^. While we did not assess whether e-cigarettes used by study respondents contained nicotine, previous analyses of these data found that 76% of participants reported that their e-cigarettes always or sometimes contained nicotine^14^. This has also been demonstrated across secondary school children (aged 13–18 years) in New Zealand whereby 80% of regular users and 90% of weekly users of e-cigarettes, reported using nicotine containing devices^15^. The introduction of ‘pod’ devices onto the market in 2015, which typically do not offer nicotine-free options, is one possible cause of youth using nicotine-containing e-cigarettes^16,17^. Students who use popular pod devices such as JUUL may not be aware of the amount of nicotine they are consuming. For example, a 2019 US survey study of high school students reported poor understanding of the levels of nicotine contained in JUUL in addition to more than one-third of participants being unaware whether a JUUL was an e-cigarette or not^18^.

Age is also a significant factor regarding e-cigarette and smoking behavior. As demonstrated in previous^8^ and by our current survey data, 18-year-olds are significantly more likely to report ever smoking and e-cigarette use compared with younger age groups. We found that use of e-cigarettes by others within the home was associated with e-cigarette use among youth, and the same was true for tobacco smoking. This is not surprising and is a well-known risk factor for smoking behavior in young people, observed in a range of settings^19-22^. We also found that reporting exposure to promotion of e-cigarettes was linked to ever use of these products, which is a concern. A 2018 RCT explored the effect of advertisements designed to attract youth on tobacco, and e-cigarette attitudes and susceptibility among 417 non-smoking adolescents^23^. It was found that participants exposed to the advertisements designed to attract youth were almost seven times more likely to select e-cigarettes in tests of choice, than the control group. This study also found that susceptibility to e-cigarettes increased even among participants exposed to advertisements designed to have low levels of appeal to youth^23^. Similarly, a recent systematic review found that pod-based e-cigarettes have been targeting young people with social media advertising, with minimal information on the health risks and usage as a smoking cessation tool compared to other e-cigarette devices^24^. These findings underscore the need to strengthen enforcement of the rules on advertising, promotion and enforcement, and review whether they need further strengthening, particularly for popular pod-based devices and social media marketing spaces.

Disposable e-cigarette use in the US and UK is growing, recent data from a cross-sectional survey of adults in Great Britain concluded that use of disposable e-cigarettes grew significantly in 2022, predominantly by those aged 18 years^25^. We found that among ever e-cigarette users, use of disposables was reported by more than half of respondents, more commonly among females and youth aged 18 years. More research is needed to understand the reasons and risk factors for use of disposable e-cigarettes among younger populations.

### Limitations

Limitations of the current study include the cross-sectional survey study design, thus we cannot infer causality or explain trajectories of tobacco smoking, e-cigarette use and quitting of products. It is plausible that some e-cigarette use in this population is among tobacco smokers attempting to quit, or may replace trying smoking, and further research into these issues is needed. Importantly, the study sample was designed to be representative of the Great Britain population and these results add to the ongoing surveillance of smoking and e-cigarette use among the UK’s younger population, which is vital for understanding and informing tobacco control policy. There may also be some confusion around what does and does not constitute an e-cigarette. The survey, however, did clarify to participants that e-cigarettes are ‘sometimes called vapes, shisha pens or electronic cigarettes’. The study was conducted in Great Britain, thus these results may not necessarily be extrapolated to other settings, and the fact that we did not assess the presence or absence of nicotine in the e-cigarettes used, nor the specific ingredients included.

## CONCLUSIONS

Rates of e-cigarette use among young people are now higher than smoking, though the vast majority of those who are regular e-cigarette users also smoke or have done so in the past. Measures are needed to reduce the appeal of these devices to young people as well as enforcement of laws to prevent illegal sales and marketing.

## Data Availability

The data supporting this research are available from the authors on reasonable request.
